# Aging Predicts Decline in Explicit and Implicit Memory: A Life-Span Study

**DOI:** 10.1177/0956797620927648

**Published:** 2020-07-31

**Authors:** Emma V. Ward, Christopher J. Berry, David R. Shanks, Petter L. Moller, Enida Czsiser

**Affiliations:** 1 Psychology Department, Middlesex University; 2 School of Psychology, University of Plymouth; 3 Division of Psychology and Language Sciences, University College London

**Keywords:** aging, explicit memory, implicit memory, priming, recognition, open data

## Abstract

Explicit memory declines with age, but age effects on implicit memory are debated. This issue is important because if implicit memory is age invariant, it may support effective interventions in individuals experiencing memory decline. In this study, we overcame several methodological issues in past research to clarify age effects on implicit memory (priming) and their relationship to explicit memory (recognition, source memory). We (a) recruited a large life-span sample of participants (*N* = 1,072) during a residency at the Science Museum in London, (b) employed an implicit task that was unaffected by explicit contamination, and (c) systematically manipulated attention and depth of processing to assess their contribution to age effects. Participants witnessed a succession of overlapping colored objects, attending to one color stream and ignoring the other, and identified masked objects at test before judging whether they were previously attended, unattended, or new. Age significantly predicted decline in both explicit and implicit memory for attended items.

As the proportion of the global population over 65 years of age rises, efforts to clarify age-related changes in memory become increasingly urgent. Explicit memory—the conscious retrieval of previously studied information—declines with age. However, age effects on implicit memory—changes in task performance due to prior exposure to stimuli that do not require conscious recollection—remain debated. Discrepancy in the literature means that no conclusion surrounding age effects on implicit memory can be drawn. This issue is important because if implicit memory is age invariant, it may support effective interventions in individuals experiencing memory decline. For instance, errorless learning, mediated by implicit memory, may help older adults learn new face–name pairings (Haslam, Hodder, & Yates, 2011).

Reviews have attributed inconsistencies between studies to a range of methodological factors (Fleischman, 2007; Mitchell & Bruss, 2003; Ward & Shanks, 2018), but no study has leveraged critical recommendations to provide conclusive evidence as to whether implicit memory declines or remains stable with age.

## Samples, Power, and Reliability

A substantial body of research suggests that, despite significant reductions in explicit memory, implicit memory remains stable over the life span and is similar in young and older adults (for a review, see Ward & Shanks, 2018). However, sample sizes have varied considerably, and small but real age differences may have gone undetected because of low statistical power. In cross-sectional studies reporting no reliable age difference in priming (a common measure of implicit memory), priming scores have usually been numerically lower in older than in younger adults, and a meta-analysis by La Voie and Light (1994) uncovered a significant age effect.

This issue is exacerbated by inherent differences in task sensitivity to age effects. Comparisons are frequently made between recognition and word-stem completion as explicit and implicit tasks, but Buchner and Wippich (2000) showed that word-stem completion has statistically lower reliability than recognition, and this could explain age-differential patterns (see also West, Vadillo, Shanks, & Hulme, 2018). The goal of a recognition task (to discriminate between studied and new items) is highly constrained, whereas word-stem completion, which involves completing stems (e.g., HO___) with the first word that comes to mind, is less so. This flexibility leads to response variability and increased error variance, making it difficult to detect small effects, and may explain why many prior studies have uncovered significant age differences in explicit but not implicit memory. Word-stem and word-fragment completion are common implicit tests, yet poor reliability may mask a genuine age-related decline in implicit memory. Buchner and Wippich found that a perceptual-identification task had greater reliability than word-stem completion and equivalent reliability to that of recognition. Perceptual identification, like recognition, has a constrained goal (to quickly identify items), and response variability is further reduced by its speeded nature.

## Processing and Task Characteristics

Depth of processing during encoding has varied across studies, with some encouraging deep conceptual processing and others encouraging shallow perceptual processing (see Mitchell & Bruss, 2003). However, older adults are impaired in elaborative and conceptual encoding (e.g., Rybash, 1996), which may explain greater age effects in studies involving conceptual processing (e.g., Ward, Berry, & Shanks, 2013) and smaller or absent age effects in studies involving perceptual processing (e.g., Soldan, Hilton, Cooper, & Stern, 2009).

Moreover, sometimes items are presented in an unattended stream or as irrelevant information during encoding (e.g., Gopie, Craik, & Hasher, 2011; Ward, 2018). However, older individuals experience greater difficulty with focused attention and filtering of irrelevant information (Healey, Campbell, & Hasher, 2008), so processing may differ. Attention and depth of processing are thus key potential moderators of age effects on implicit memory, yet they have never been systematically manipulated to gain a clear understanding of these effects.

## Explicit Contamination

Some implicit tasks may be susceptible to contamination by explicit memory strategies. This is a significant issue when it comes to aging; reduced priming with age could reflect the use of explicit strategies that are more beneficial to young adults. Mitchell (1995) reported that age differences in implicit memory disappeared when data were adjusted for explicit contamination, and Russo and Parkin (1993) found an age effect that disappeared when explicit memory was equated between groups (see also Geraci & Barnhardt, 2010). Importantly, the meta-analysis by La Voie and Light (1994), which uncovered a significant age effect on implicit memory, did not account for explicit contamination.

Numerous recommendations to circumvent explicit contamination have been put forward, and these have largely been centered on reducing awareness of the connection between study and test (MacLeod, 2008). However, a valuable method for studying the relationship between explicit and implicit memory is to measure them concurrently using the continuous-identification-with-recognition (CID-R) task (e.g., Stark & McClelland, 2000). On each trial of the CID-R task, a word or an object is identified prior to a recognition judgment. Indices of explicit and implicit memory for each item are captured within a few hundred milliseconds of one another, making them more suitable for comparison than measures sampled in separate experimental phases involving a delay.

In the CID-R paradigm, participants are aware that studied items are presented at test, and they could feasibly attempt to use an explicit strategy. However, there is evidence that priming on this task is unaffected by explicit contamination. For instance, Brown, Jones, and Mitchell (1996) reported no difference in priming when identification and recognition were measured concurrently trial by trial relative to in separate experimental phases. Brown, Neblett, Jones, and Mitchell (1991; see also Mitchell & Bruss, 2003) found no difference in priming on picture- and word-naming tasks between participants who witnessed studied and new items in separate blocks (and were informed which block contained which type of item) and participants who saw studied and new items interspersed. Ward et al. (2013) replicated the above finding of no difference in priming when the identification task was presented alone and when concurrent recognition judgments were elicited (Experiment 2). They also found that identification-task performance was not enhanced by informing participants whether the next item to appear was previously studied or new, nor was it hindered when such explicit cues were incorrect (Experiments 3a and 3b). Thus, explicit processing does not appear to affect priming in the CID-R paradigm, and this may be because identification is accomplished too quickly for the engagement of effortful explicit strategies (MacLeod, 2008).

## The Current Investigation

In this study, we aimed to overcome the issues described above to clarify age effects on implicit memory (priming) and their relationship to explicit memory (recognition, source memory). The study was highly powered and employed a CID-R task that evidence suggests is unaffected by explicit contamination. Attention and depth of processing were manipulated to reveal their contribution to age effects, and source memory (retrieval of contextual detail associated with an item’s presentation) was captured as an additional explicit measure, as the relationship among priming, recognition, and source memory in aging has never been examined in this context. Evidence for preserved implicit memory with age would be stable priming with age (supported by Bayesian analyses providing evidence in favor of the null hypothesis) coupled with reliable reductions in explicit memory. To reveal the central finding, we found that age predicted decline in both explicit and implicit memory for attended items.

## Method

The study took place during a residency at the Science Museum in London, where adolescents through older adults were recruited to map memory changes across the life span. This is an important departure from studies that habitually compare relatively small samples of young (~18–30 years) and older (~65+ years) adults. Participants were shown overlapping line drawings of objects colored in cyan and magenta, attending to one color and ignoring the other. They judged whether objects were angular or rounded (shallow processing) or natural or manufactured (deep processing) before completing a CID-R task in which they identified a masked object on each trial before judging whether it was previously presented in cyan, previously presented in magenta, or new.

### Participants and design

Many prior studies have used inappropriately small sample sizes, and small effects may have gone undetected because of low statistical power. Further, most studies have compared the performance of small groups of young and older adults, whereas this study recruited a large life-span sample. Over the course of a 6-week residency at the Science Museum, 1,072 visitors (448 male) between the ages of 12 and 82 years volunteered to take part. Ethical approval was granted by the Middlesex University Research Ethics Committee, and all participants provided informed consent, including parent or guardian consent for those under 16. Participants were required to be fluent in English and have normal or corrected-to-normal vision, no color blindness, and no history of memory problems. There was no upper age limit, but older participants were required to be healthy and free of dementia. Twenty-one participants (9 adolescents, 2 young, 3 mid-young, 1 middle, 1 mid-older, and 5 who gave no age) were excluded because of missing information or accuracy levels below 80% in the identification task (see the Procedure section). The final sample consisted of 1,051 participants (443 male) between the ages of 12 and 82 years (*M* = 29.36, *SD* = 14.31).

Because of the open environment and the time restrictions imposed by the museum, it was necessary to keep background tests to a minimum. Information was collected on age, sex, years of education, visual acuity, intellectual functioning (using the Wechsler Test of Adult Reading, WTAR, Wechsler, 2001, for participants 16 years of age and above), and processing speed. No formal assessment of cognitive impairment (e.g., Mini-Mental State Exam) could be performed on older participants, but we can be confident that all were free of cognitive impairment because (a) they were asked to confirm this eligibility requirement when providing consent, and (b) there were no outliers or anomalies in the test data or WTAR scores to suggest abnormal function. Sample characteristics are provided in Table 1, in which participants are segregated into six life-span groups: adolescents (12–17 years), young adults (18–24 years), mid-young adults (25–34 years), middle adults (35–49 years), mid-older adults (50–64 years), and older adults (65–82 years).

**Table 1.** Participant Characteristics

Variable	Adolescents (*n* = 211)	Young adults (*n* = 291)	Mid-young adults (*n* = 261)	Middle adults (*n* = 170)	Mid-older adults (*n* = 83)	Older adults (*n* = 35)
Age range (years)	12–17	18–24	25–34	35–49	50–64	65–82
Mean age (years)	14.67 (1.89)	21.07 (2.00)	28.83 (2.98)	41.42 (4.19)	55.30 (4.36)	70.60 (4.40)
Gender (*n*)						
Male	72	122	123	78	32	16
Female	139	169	138	92	51	19
Processing condition (*n*)						
Deep	118	152	138	90	45	18
Shallow	93	139	123	80	38	17
Mean years of education^a^	10.51 (2.31)	15.60 (2.68)	17.45 (3.09)	17.98 (3.87)	17.88 (6.05)	16.26 (5.11)
Mean WTAR score^a^	38.51 (6.23)	39.91 (7.51)	42.06 (6.79)	43.18 (6.35)	42.46 (7.15)	45.94 (3.64)
Mean visual acuity^a^	37.13 (12.17)	37.36 (13.26)	34.94 (7.04)	40.89 (16.27)	42.25 (11.68)	49.54 (27.89)
Mean processing speed (ms)^a^	2,412 (461)	2,217 (449)	2,225 (453)	2,414 (559)	2,580 (638)	2,870 (640)

Note: Standard deviations for all mean values are given in parentheses. The Wechsler Test of Adult Reading (WTAR; Wechsler, 2001), in which participants are asked to pronounce uncommon English words (maximum score: 50), is an assessment of intellectual functioning. This was administered to participants 16 years old and over; thus, the score for adolescents is based on 97 participants who met this criterion. The mean for mid-older adults excludes one participant with a missing score. Visual acuity was measured using the Near Vision Test Card (http://www.i-see.org/block_letter_eye_chart.pdf), viewed at a distance of 16 in. Scores can range from 16 (highest acuity) to 160 (lowest acuity). One adolescent with a score of 14 was not included. Processing speed was indexed as the mean of the baseline (new-item) identification times in the continuous-identification-with-recognition (CID-R) task.

a There was a significant main effect of age group for these variables (*p*s < .001). Bonferroni-corrected follow-up comparisons indicated significant differences in years of education (adolescents vs. all other groups; young adults vs. all groups apart from older adults), visual acuity (adolescents vs. mid-older adults and older adults; young adults vs. mid-older and older adults; mid-young vs. middle adults, mid-older adults, and older adults), WTAR score (adolescents vs. all groups apart from young adults; young adults vs. all groups apart from adolescents; mid-young vs. older adults), and processing speed (adolescents vs. all groups apart from mid-young and middle adults; young adults vs. all groups apart from mid-young adults; mid-young vs. all groups apart from young adults; middle adults vs. all groups apart from adolescents and mid-older adults; older adults vs. all groups apart from mid-older adults).

Priming (speed of perceptual identification), recognition, and source memory were assessed using a CID-R task following a separate study phase in which attention was manipulated within participants (attended items, unattended items) and depth of processing was manipulated between participants (shallow processing, deep processing).

### Stimuli

**Fig. 1.**
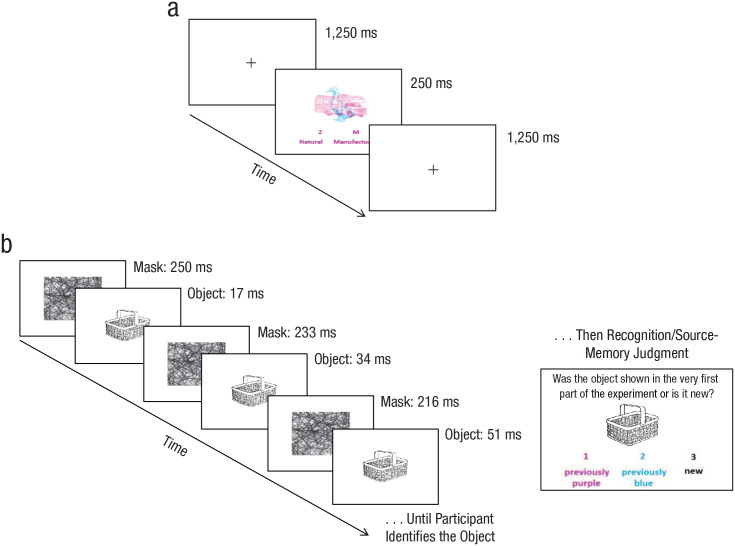
Example trials from the study phase and the continuous-identification-with-recognition (CID-R) task. In the study phase (a), participants were shown a stream of overlapping objects, one colored cyan and the other colored magenta, and asked to attend to one color stream and ignore the other (counterbalanced across participants). On each trial, participants judged whether the attended item was natural or manufactured (deep-processing condition) or angular or rounded (shallow-processing condition). The text color of the response cues (“Z” or “M”) served as a reminder of the color stream to attend to (magenta in this example). In the CID-R task (b), an object—old (attended or unattended) or new—gradually clarified from a background mask, and participants identified the object as quickly as possible (priming measure) before making a recognition/source-memory judgment (explicit measures). During the clarification procedure, the background mask was initially presented for 250 ms prior to a flash of the object for 17 ms. Presentations of the mask and object were then alternated, with the object duration increasing by 17 ms and the mask duration decreasing by 17 ms each alternate cycle, with the result that the object gradually became clearer. A key press ended the clarification procedure, at which point the object disappeared, and the participant’s identification RT was captured. The participant then typed the object name into a box before the object was presented again for the recognition/source-memory judgment.

The stimuli were a subset of the 260 line drawings of everyday objects created by Snodgrass and Vanderwart (1980). Highly similar items (e.g., blouse, jacket) were removed, leaving 245 objects. Approximately half were naturally occurring items, and half were manufactured. Objects were 240 × 240 pixels in size and were presented in the center of a white background screen. Forty were presented in the study phase, 20 colored in magenta and 20 in cyan, with approximately equal brightness and luminance. On each trial, two objects—one magenta and one cyan—overlapped (Fig. 1a). Eighty objects in total were presented at test, 40 from the study phase (20 attended, 20 unattended) and 40 new items. Objects were presented in black at test. Each object was randomly assigned to serve as an attended, an unattended, or a new item, and a different random assignment was used for each participant.

The mask used in the identification task (Fig. 1b) was 400 × 400 pixels in size and was created using a script that randomly superimposed lines and arcs of a similar thickness onto the lines of objects in the stimulus set.

### Procedure

The experiment took place within the Live Science space at the Science Museum in South Kensington, London. There were five desktop PCs with a screen resolution of 1,280 × 1,024 pixels. Participants performed the experiment individually, but up to five could take part at one time. An adjustable screen surrounding the area gave privacy and ensured that any waiting participants could not view the experiment. Guidelines for the residency stated that the procedure should not exceed 30 min, so the experimental task was designed to take 20 to 25 min, and the remaining 5 to 10 min were spent collecting background information, including age, sex, years of education, visual acuity, and intellectual functioning (using the WTAR; Table 1). This was done prior to the experimental task and after the eligibility check.

The experiment was programmed using MATLAB 2016b (The MathWorks, Natick, MA). Viewing distance was approximately 50 cm. During the study phase (Fig. 1a), participants witnessed a stream of overlapping object pairs. One of the objects was presented in magenta, and the other was presented in cyan. Participants were told that the objects would be presented briefly and that they should attend to one color (either magenta or cyan) and ignore the other. The attended color was randomly assigned across participants and collapsed for analysis. Each object pair was presented for 250 ms, followed by a black fixation cross presented for 1,250 ms. The duration of the interstimulus interval was chosen to allow time for a response on each trial. The response depended on which depth-of-processing manipulation participants had been randomly assigned to receive. In the *deep-processing condition*, participants decided whether the attended item was natural or manufactured, and in the *shallow condition*, they determined whether it was angular or rounded. Using the Z and M keys, participants were instructed to respond as quickly as possible on the basis of their first impression. The response cues “Z = natural; M = manufactured” or “Z = angular; M = rounded” remained on the screen at all times in a font color that matched the attended stream of objects. There were 8 practice trials prior to the 40 experimental trials.

Following the study phase, there was a retention interval of approximately 3 min while participants read instructions for the CID-R task. Each trial consisted of a speeded object identification followed by a recognition/source-memory judgment. Forty randomly chosen old objects from the study phase (20 attended, 20 unattended) appeared at test, along with 40 new objects, all of which were presented in black on a white background. Participants were informed that, on each trial, they would first have to identify an object that would be masked and difficult to make out. They were informed that the object would appear to gradually emerge and that their task was to press the enter key as soon as they could identify it. Speed was emphasized, but participants were asked to be as accurate as possible.

Each trial ran as follows (see Fig. 1b). The mask was presented for 250 ms, followed by an object for 17 ms (with one screen refresh at 60 Hz) and then the mask again for 233 ms, forming a 250-ms block. The block presentations continued with the object presentation increasing by 17 ms on each alternate cycle and the mask duration decreasing by the same amount, with the result that the object appeared to gradually become clearer. Identification time was the moment when participants pressed the enter key, at which time the object disappeared, and participants were prompted to type their response (e.g., “basket”) into a box. If the enter key had not been pressed after 7,500 ms (i.e., when the object was fully displayed), the trial was discarded, and the cue “Please try to be faster on the next trial” appeared for 1,000 ms.

After identifying the object, participants judged whether it was shown in the study phase and, if so, in what color—the color that they had attended to or the color that they had ignored. The object was presented once more (in full view) along with the instruction, “Was the object shown in the very first part of the experiment or is it new?” (1 = *previously purple*, 2 = *previously blue*, 3 = *new*). Participants responded by pressing a number key. They were informed that half of the objects had been presented previously (an equal number of attended and unattended items) and half were new. No time limit was imposed. Following a response, a fixation cross was presented for 500 ms prior to the next trial. At the end of the experiment, participants received on-screen feedback with their average response times (RTs) and recognition scores (in percentages) for attended, unattended, and new objects.

## Results

An alpha level of .05 was used for all tests, and *t* tests were two-tailed. Effect sizes are reported as η_*p*_^2^s for significant analysis-of-variance (ANOVA) effects and as Cohen’s *d*s for *t* tests.

### Study phase

Trials on which no key was pressed or the RT was less than 200 ms were excluded. One older participant made no key presses in the study phase and was not included in this analysis. Mean classification RTs did not significantly differ between the deep (*M* = 701 ms, *SE* = 6) and shallow (*M* = 700 ms, *SE* = 7) conditions, *t*(1048) = 0.02, *p* = .985, *d* = 0.001, 95% confidence interval (CI) = [−0.12, 0.12].

### Priming

**Fig. 2.**
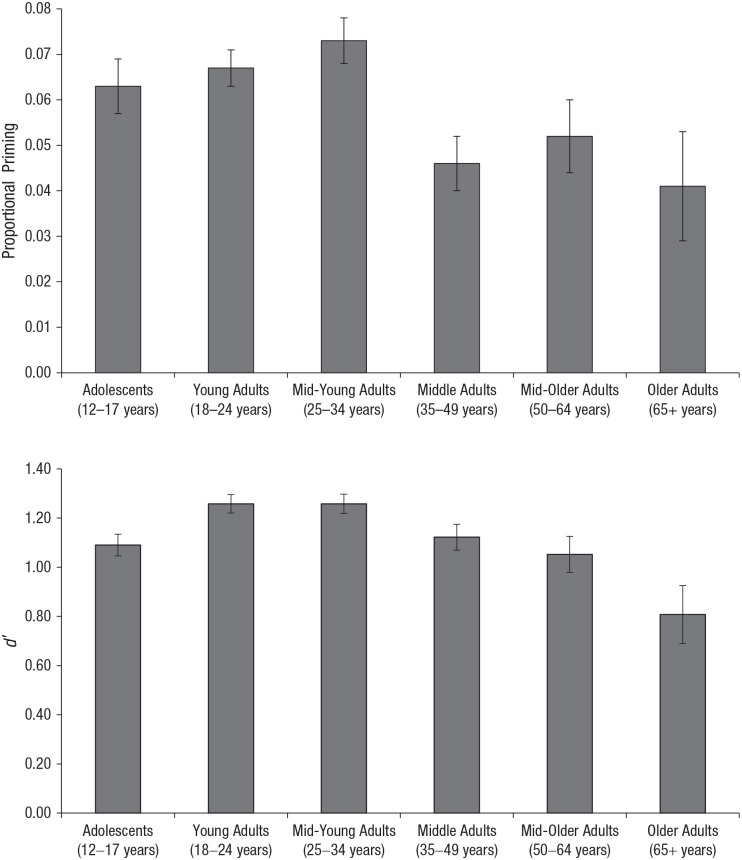
Mean proportional priming (top) and mean *d*′ score (bottom) in each age group for attended items, collapsed across depth-of-processing condition. Error bars indicate standard errors of the mean.

We excluded trials associated with incorrect identifications and with RTs less than 200 ms or more than 3 standard deviations from the mean (4.90% of trials). Table 2 reports identification rates and RTs for attended, unattended, and new items. Priming was calculated by subtracting each participant’s mean old-item RT (attended and unattended) from his or her mean new-item RT, expressed in proportion to their mean baseline (new-item) RT: (RT new – RT old)/RT new (see Fig. 2). A proportional measure is deemed most suitable for age comparisons, because slower baseline responding in older than in young adults can artificially elevate priming when an RT difference score is used (e.g., Faust, Balota, Spieler, & Ferraro, 1999).

**Table 2.** Perceptual Identification and Priming Across Age Groups

Measure and item type	Adolescents (12–17 years)	Young adults (18–24 years)	Mid-young adults (25–34 years)	Middle adults (35–49 years)	Mid-older adults (50–64 years)	Older adults (65+ years)
Identification rate (%)	93.73 (3.87)	95.18 (3.82)	95.69 (3.14)	95.75 (5.91)	95.29 (3.85)	94.68 (3.16)
Deep-processing condition						
Mean old-item RT (ms)						
Attended	2,227 (417)	2,081 (489)	2,019 (436)	2,351 (573)	2,545 (680)	2,579 (684)
Unattended	2,351 (433)	2,223 (497)	2,197 (469)	2,410 (546)	2,676 (645)	2,673 (617)
Mean new-item RT (ms)	2,396 (449)	2,233 (461)	2,208 (436)	2,446 (553)	2,695 (651)	2,679 (610)
Proportional priming						
Attended	.07 (.08)	.07 (.08)	.08 (.08)	.04 (.08)	.06 (.07)	.04 (.06)
Unattended	.01 (.09)	.01 (.08)	.00 (.08)	.01 (.08)	.00 (.07)	.00 (.05)
Shallow-processing condition						
Mean old-item RT (ms)						
Attended	2,300 (526)	2,056 (420)	2,105 (454)	2,254 (576)	2,337 (607)	2,944 (599)
Unattended	2,387 (464)	2,176 (445)	2,234 (484)	2,349 (555)	2,418 (632)	3,021 (521)
Mean new-item RT (ms)	2,433 (478)	2,199 (437)	2,243 (473)	2,378 (566)	2,445 (602)	3,072 (625)
Proportional priming						
Attended	.06 (.08)	.06 (.07)	.06 (.08)	.05 (.07)	.04 (.07)	.04 (.06)
Unattended	.02 (.07)	.01 (.08)	.00 (.07)	.01 (.06)	.01 (07)	.01 (.08)

Note: Standard deviations are given in parentheses. Identification rate is the percentage of continuous-identification-with-recognition (CID-R) trials remaining after screening. Trials associated with incorrect identifications or response times (RTs) less than 200 ms or more than 3 standard deviations from the mean were excluded. Proportional priming was calculated as (RT new – RT old)/RT new.

A 6 (age) × 2 (attention) × 2 (depth of processing) mixed ANOVA revealed a significant main effect of attention on priming, *F*(1, 1039) = 172.02, *p* < .001, η_*p*_^2^ = .142, and a significant interaction between attention and age, *F*(5, 1039) = 3.84, *p* = .002, η_*p*_^2^ = .018. No other main effects or interactions were significant (*p*s > .05). The Attention × Age interaction suggests a statistical age effect for attended but not unattended items. Priming for attended items was statistically above zero in all age groups (*p*s < .008, *d*s > 1.38, Bonferroni-adjusted alpha), but priming for unattended items was not significant in any group apart from adolescents, *t*(210) = 2.86, *p* = .005, *d* = 0.20, 95% CI = [0.06, 0.33], so a one-way ANOVA was performed on attended items where priming was present. Given no main effect of depth of processing, *F*(1, 1039) = 0.25, *p* = .616, η_*p*_^2^ < .001, the deep and shallow conditions were collapsed. This revealed a significant main effect of age, *F*(5, 1045) = 3.59, *p* = .003, η_*p*_^2^ = .017. Confirming these findings, multiple regression with age as a continuous variable revealed a significant linear decline in priming for attended items with age, *F*(1, 1049) = 7.82, *p* = .005, *r* = −.086. No extra variance was explained by adding a quadratic component of age (Δ*R*^2^ < .001, *p* = .483). An orthogonal component with a correlation of 0 was used to overcome multicolinearity.

### Recognition and source memory

On each trial, participants judged whether an object was previously presented in blue or purple (“old” judgment) or was not previously presented (“new” judgment). For recognition-memory analyses, ratings 1 (*previously purple*) and 2 (*previously blue*) were collapsed to a single “old” judgment. For each participant, *d*′ was calculated as the *z*-transformed hit rate (proportion of old items judged old) minus the *z*-transformed false-alarm rate (proportion of new items judged old), separately for attended and unattended items (Table 3).

**Table 3.** Recognition Memory Across Age Groups

Measure and item type	Adolescents (12–17 years)	Young adults (18–24 years)	Mid-young adults (25–34 years)	Middle adults (35–49 years)	Mid-older adults (50–64 years)	Older adults (65+ years)
Deep-processing condition						
Hit rate						
Attended	.77 (.21)	.79 (.17)	.81 (.14)	.74 (.18)	.70 (.22)	.60 (.24)
Unattended	.41 (.21)	.39 (.21)	.37 (.20)	.40 (.24)	.37 (.26)	.41 (.20)
FA rate	.38 (.19)	.37 (.19)	.35 (.18)	.37 (.21)	.33 (.22)	.34 (.19)
*d*′						
Attended	1.15 (0.68)	1.26 (0.67)	1.31 (0.61)	1.10 (0.69)	1.04 (0.60)	0.74 (0.76)
Unattended	0.09 (0.41)	0.06 (0.39)	0.07 (0.42)	0.12 (0.45)	0.09 (0.38)	0.22 (0.30)
Shallow-processing condition						
Hit rate						
Attended	.75 (.21)	.84 (.13)	.76 (.19)	.73 (.23)	.68 (.24)	.58 (.31)
Unattended	.44 (.22)	.44 (.19)	.39 (.20)	.35 (.25)	.39 (.24)	.30 (.20)
FA rate	.40 (.21)	.42 (.18)	.35 (.18)	.33 (.22)	.34 (.23)	.29 (.18)
*d*′						
Attended	1.01 (0.59)	1.26 (0.63)	1.20 (0.64)	1.15 (0.67)	1.07 (0.75)	0.88 (0.64)
Unattended	0.12 (0.40)	0.07 (0.39)	0.12 (0.38)	0.05 (0.44)	0.18 (0.36)	0.08 (0.39)

Note: Standard deviations are given in parentheses. Hit rate is the mean proportion of “old” judgments to old items (attended and unattended). Responses 1 and 2 on the scale were collapsed to a single “old” judgment. False-alarm (FA) rate is the mean proportion of “old” judgments to new items. Performance (*d*′) was calculated as *z*(hit rate) − *z*(FA rate). The Snodgrass and Corwin (1988) correction was applied to hit rates and FA rates, that is, hit rate = (*n* hits + 0.5)/(*n* old + 1); FA rate = (*n* FAs + 0.5)/(*n* new + 1).

Recognition was significantly above chance (*d*′ > 0) for attended items in all age groups (Bonferroni-adjusted *p*s < .008, *d*s > 3.53) but not for unattended items in middle adults, *t*(169) = 2.54, *p* = .012, *d* = 0.20, 95% CI = [0.04, 0.35], or older adults, *t*(34) = 2.57, *p* = .015, *d* = 0.43, 95% CI = [0.08, 0.78]. A 6 (age) × 2 (attention) × 2 (depth of processing) ANOVA revealed significant main effects of attention, *F*(1, 1039) = 1,290.60, *p* < .001, η_*p*_^2^ = .554, and age, *F*(5, 1039) = 2.33, *p* = .041, η_*p*_^2^ = .011, and an interaction between the two, *F*(5, 1039) = 6.98, *p* < .001, η_*p*_^2^ = .033. There was no effect of depth of processing, *F*(1, 1039) = 0.03, *p* = .873, η_*p*_^2^ < .001, and no other interactions (*p*s > .05). A one-way ANOVA revealed a significant main effect of age for attended items, *F*(5, 1045) = 5.52, *p* < .001, η_*p*_^2^ = .026, collapsed across depth of processing. There was no main effect of age on unattended items, *F*(5, 1045) = 0.61, *p* = .694, η_*p*_^2^ = .003. As with priming, multiple regression revealed that age as a continuous variable significantly predicted a linear decline in recognition of attended items, *F*(1, 1049) = 7.19, *p* = .007, *r* = −.083. An additional 1.16% of variance was explained by adding the quadratic component of age (Δ*R*^2^ = .0116, *p <* .001).

The adolescent data were included to shed light on life-span changes in priming and recognition. For readers interested specifically in adult age differences, an analysis excluding adolescents revealed a consistent pattern. When we collapsed across depth of processing, there were significant main effects of age on recognition (*d*′), *F*(4, 835) = 5.85, *p* < .001, η_*p*_^2^ = .027, and priming, *F*(4, 835) = 4.65, *p* = .001, η_*p*_^2^ = .022 (attended items; see Fig. 2), and age as a continuous variable predicted significant linear declines in recognition, *F*(1, 838) = 19.28, *p* < .001, *r* = −.15, and priming, *F*(1, 838) = 10.94, *p* = .001, *r* = −.11.

**Fig. 3.**
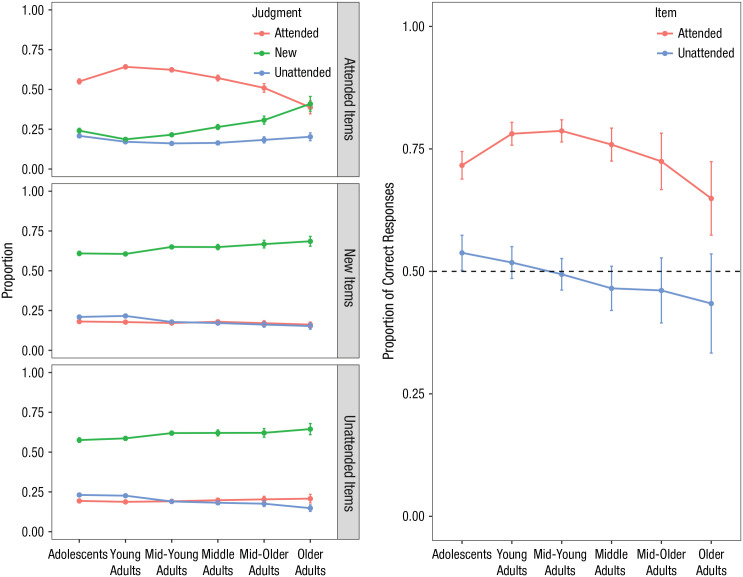
Judgment and accuracy by item type. The graphs on the left show the mean proportion of attended, unattended, and new judgments by item type, collapsed across depth-of-processing condition. Error bars indicate standard errors. The graph on the right shows the accuracy of source judgments to attended and unattended items, collapsed across depth-of-processing condition. Error bars indicate 95% confidence intervals.

Breaking down attended, unattended, and new judgments, collapsed across depth of processing, revealed that attended items tended to be correctly judged as attended rather than as unattended or new, *F*(1.67, 1746.75) = 409.18, *p* < .001, η_*p*_^2^ = .281 (Greenhouse-Geisser-adjusted degrees of freedom are reported because the assumption of sphericity was violated). This ability peaked in young adults before declining—Judgment × Age interaction: *F*(8.36, 1746.75) = 11.33, *p* < .001, η_*p*_^2^ = .051. There was an increasing tendency to judge items as new with age, and the oldest adults were unable to judge whether attended items were attended or new, *t*(34) = 0.26, *p* = .796, *d* = 0.04, 95% CI = [−0.29, 0.38] (see Fig. 3 for judgments and accuracy by item type). Both unattended and new items tended to be judged as new, *F*(1.56, 1631.69) = 789.19, *p* < .001, η_*p*_^2^ = .430, and *F*(1.46, 1537.93) = 1,257.72, *p* < .001, η_*p*_^2^ = .546, respectively, and unattended items were not accurately judged as such by any group.

### Associations between priming and recognition

Collapsing across depth of processing, we next examined whether identification RTs in the priming task varied according to whether the item was explicitly remembered (Table 4). RTs were significantly faster for items judged to be old compared with ones judged to be new, *F*(1, 1027) = 277.48, *p* < .001, η_*p*_^2^ = .213, and this was consistent across age groups (no interaction with age, *p* = .413; all paired-samples *t* tests, *p* < .001).

**Table 4.** Identification Response Times (in Milliseconds) by Recognition Judgment

Item type and measure	Adolescents (12–17 years)	Young adults (18–24 years)	Mid-young adults (25–34 years)	Middle adults (35–49 years)	Mid-older adults (50–64 years)	Older adults (65+ years)
Items judged new	2,419 (452)	2,240 (445)	2,245 (462)	2,427 (568)	2,595 (649)	2,893 (645)
Items judged old	2,307 (464)	2,100 (455)	2,095 (439)	2,298 (536)	2,484 (643)	2,745 (618)
Old items						
Attended hits	2,231 (487)	2,028 (448)	2,015 (442)	2,256 (606)	2,405 (657)	2,644 (580)
Unattended hits	2,318 (506)	2,133 (499)	2,136 (518)	2,317 (553)	2,517 (679)	2,828 (675)
Attended misses	2,386 (601)	2,268 (616)	2,211 (581)	2,454 (690)	2,647 (864)	2,879 (779)
Unattended misses	2,411 (456)	2,230 (487)	2,251 (450)	2,401 (600)	2,609 (682)	2,899 (676)
New items						
False alarms	2,367 (478)	2,162 (498)	2,166 (478)	2,344 (569)	2,588 (709)	2,816 (734)
Correct rejections	2,431 (478)	2,247 (446)	2,248 (471)	2,442 (574)	2,587 (636)	2,892 (641)

Note: Results are collapsed across depth-of-processing condition. Standard deviations are given in parentheses.

Identification RTs were also analyzed according to whether the recognition judgment was a hit, miss, false alarm, or correct rejection. RTs significantly varied according to recognition judgment, *F*(3.58, 3303.28) = 54.86, *p* < .001, η_*p*_^2^ = .056, and there was no interaction with age (*p* = .862; all Greenhouse-Geisser adjusted). Collapsed across age, identification RTs were fastest overall for attended items that were recognized (attended hits: *M* = 2,151, *SD* = 528), and slowest for correctly rejected new items (*M* = 2,364, *SD* = 525), and this difference was significant, *t*(1029) = 26.61, *p* < .001, *d* = 0.83, 95% CI = [0.76. 0.90]. Attended hits were associated with significantly faster identification times compared with attended misses, *t*(957) = 13.82, *p* < .001, *d =* 0.45, 95% CI = [0.38, 0.51], but—revealing no evidence of priming for unrecognized objects—RTs for misses did not differ from RTs for correct rejections (attended misses: *M* = 2,361, *SD* = 518; unattended misses: *M* = 2,352, *SD* = 548; correct rejections: *M* = 2,364, *SD* = 525; *p*s = .278 and .157, respectively). These clear associations between priming and recognition are consistent with previous findings from the CID-R task (Berry, Shanks, Speekenbrink, & Henson, 2012).

## Discussion

In this study, we aimed to clarify age effects in implicit memory and their relationship with explicit memory by overcoming several issues that have compromised past research. We (a) used a large life-span sample rather than a mere comparison of young and older participants, (b) employed an implicit task that evidence suggests is unaffected by explicit contamination, (c) used a seminaturalistic setting, and (d) directly manipulated attention and depth of processing (factors that have varied in prior studies) to reveal their contributions to age effects. The data revealed age-related declines in both implicit and explicit memory. Age effects were present for attended items but not for unattended items, for which performance was no greater than chance in the majority of cases. Age predicted a decline in explicit and implicit memory for attended items, with a quadratic trend in recognition indicating that it increased up to mid-young adulthood before declining. The ability to correctly judge attended items as attended rather than unattended or new peaked in young adults before declining, and older adults were unable to judge whether attended items were attended or new. There was no priming for items that were not recognized, and this was consistent across age groups.

Findings in relation to explicit memory are consistent with a body of literature. Nilsson (2003) reported an increase in explicit memory up to 25 to 30 years of age before gradual decline. A progressive decline has been shown longitudinally (e.g., Davis, Trussell, & Klebe, 2001; Fleischman, Wilson, Gabrieli, Bienias, & Bennett, 2004; Hultsch, Hertzog, Small, McDonald-Miszczak, & Dixon, 1992), and numerous cross-sectional studies show poorer performance in older compared with younger adults on recall and recognition tests (see Jelicic, Craik, & Moscovitch, 1996; Kausler, 1994).

In a field replete with contradictory findings surrounding age effects on implicit memory, this study provides much-needed clarification by addressing prominent issues and uncovering evidence of decline that qualitatively mirrors that in explicit memory. Some prior studies have reported reductions in implicit memory with age on tests of word-stem completion and perceptual identification (e.g., Abbenhuis, Raaijmakers, Raaijmakers, & Van Woerden, 1990; Hultsch, Masson, & Small, 1991; Ward et al., 2013), but the present study controlled for the possibility that the effect is mediated by explicit contamination or by differences in processing or attention.

Although the qualitative patterns of change in explicit and implicit memory are consistent, the decline in implicit memory is smaller than that in explicit memory. This is consistent with the meta-analysis by La Voie and Light (1994) and is likely a function of differences in task sensitivity. Implicit tasks are generally associated with greater variability than explicit tasks (e.g., Buchner & Wippich, 2000; West et al., 2018), meaning that it is more difficult to statistically detect effects in the former. Indeed, a single-system computational model developed by Berry and colleagues predicts larger effects on recognition than on priming by assuming that the error variance associated with the priming task is greater than that associated with recognition (e.g., Berry et al., 2012). Variability of baseline (new-item) RTs was highest in the oldest group in the present study, and this may have been exacerbated by the relatively small size of this group.

The observed age-related decline would benefit from validation in a longitudinal design to determine within-individual changes in memory over time. Longitudinal studies are less common in the literature but have largely revealed reductions in explicit memory with age coupled with null changes in priming (e.g., Davis et al., 2001; Fleischman, Wilson, Gabrieli, Bienias, & Bennett, 2004). These studies are susceptible to the same problems reviewed here and may have failed to statistically detect small changes in priming. We conjecture that a similar longitudinal study—although extremely difficult to accomplish on a similar scale to the present study—would be likely to expose a consistent pattern of decline in explicit and implicit memory.

There was no effect of depth of processing. This was manipulated in a manner that has produced effects on explicit memory in the past, albeit not unfailingly (see Intraub & Nicklos, 1985). However, most prior studies have used words as stimuli and free recall as the task. There is also evidence that semantic processing can occur with extremely brief presentations (e.g., Potter, Wyble, Hagmann, & McCourt, 2014), so exposure time in this study may have been sufficiently long to enable deep processing in both conditions. Indeed, classification times were similar in the deep and shallow conditions, suggesting equivalent processing. The observations suggest that differential processing during encoding (i.e., deep vs. shallow, as may naturally occur in young adults vs. older adults) is an unlikely mediator of age effects in implicit memory. Rather, variations in the magnitude of age differences are likely due to a combination of issues with power and task reliability.

To conclude, this study clarifies the effect of age on implicit memory, delivering robust evidence for a decline that qualitatively mirrors that in explicit memory. This has significant implications for an aging population, suggesting limits to the utility of implicit memory for supporting interventions in individuals experiencing memory decline. The findings also hold implications for our theoretical understanding of the organization of memory, suggesting that explicit and implicit memory do not operate independently but are driven by a single underlying system (Berry et al., 2012).

## Supplemental Material

Ward_OpenPracticesDisclosure_rev – Supplemental material for Aging Predicts Decline in Explicit and Implicit Memory: A Life-Span Study Click here for additional data file. Supplemental material, Ward_OpenPracticesDisclosure_rev for Aging Predicts Decline in Explicit and Implicit Memory: A Life-Span Study by Emma V. Ward, Christopher J. Berry, David R. Shanks, Petter L. Moller and Enida Czsiser in Psychological Science

## Transparency

*Action Editor:* Caren Rotello

*Editor:* D. Stephen Lindsay

*Author Contributions*

E. V. Ward developed the study concept. All authors contributed to the study design. C. J. Berry and E. V. Ward designed the experimental task, which was programmed by C. J. Berry. Data collection and data screening were managed by E. Czsiser and P. L. Moller under the supervision of E. V. Ward. Data were analyzed by E. V. Ward and C. J. Berry, and all authors contributed to their interpretation. E. V. Ward drafted the manuscript, and critical revisions were provided by C. J. Berry and D. R. Shanks. All authors approved the final version of the manuscript for submission.

**Open Practices:** All data have been made publicly available via the Open Science Framework and can be accessed at https://osf.io/d6j4u/. The design and analysis plans for this study were not preregistered. The complete Open Practices Disclosure for this article can be found at http://journals.sagepub.com/doi/suppl/10.1177/0956797620927648. This article has received the badge for Open Data. More information about the Open Practices badges can be found at http://www.psychologicalscience.org/publications/badges.
